# Evaluation of urine protein to creatinine ratio in sighthound breeds

**DOI:** 10.17221/74/2024-VETMED

**Published:** 2024-12-27

**Authors:** Simona Kovarikova, Denisa Jurujova, Kristyna Panykova, Jana Blahova

**Affiliations:** Department of Animal Welfare and Protection and Veterinary Public Health, University of Veterinary Sciences, Brno, Czech Republic

**Keywords:** Greyhound, proteinuria, Spanish Greyhound, urinalysis, Whippet

## Abstract

The breed can influence the results of haematological and biochemical blood tests, with sighthounds traditionally mentioned. It may also affect certain urinary parameters. This study aimed to compare urinary protein and creatinine concentrations and their ratio (UPC, urine protein to creatinine ratio) between sighthounds and non-sighthounds and to evaluate these parameters in various sighthound breeds. Urine samples from clinically healthy dogs were collected via normal voiding, representing both sighthound and non-sighthound breeds. The protein and creatinine concentrations in the urine samples were determined, and their ratio was subsequently calculated. A total of 191 urine samples from sighthounds and 90 urine samples from non-sighthound breeds used as a control group were evaluated in the study. In sighthounds, significantly lower urinary protein concentration (248.8 mg/l and 299.8 mg/l, respectively; *P* = 0.045) and significantly higher urinary creatinine concentration (23.0 mmol/l and 17.5 mmol/l, respectively; *P* = 0.000) and lower UPC values (0.13 and 0.18, respectively; *P* = 0.000) were observed in comparison to the entire control group. The UPC values were found to be significantly lower in Greyhounds and Spanish Greyhounds compared with non-sighthounds. Although statistically significant changes were identified, they are unlikely to be of great clinical importance.

The results of haematological and biochemical examinations may be influenced by various factors, including age, sex, breed, or weight. The living environment of the animal as well as its lifestyle, diet, the method of sample collection and the time of year when the sample is obtained may also have an effect. It has recently been demonstrated that the breed represents a significant factor in dogs (Gommeren et al. 2006; Xenoulis et al. 2007; Rosset et al. 2012; Ruaux et al. 2012; Torres et al. 2014; Conrado et al. 2014; Misbach et al. 2014; Rortveit et al. 2015). Currently, the canine population numbers more than 300 dog breeds, exhibiting differences not only in appearance but in potentially differing haematological and biochemical phenotypes. Thus, the concept of uniform reference ranges for the entire canine population seems no longer valid; certain breeds require further necessary specification (Sharkey et al. 2009; Nielsen et al. 2010; Lavoue et al. 2013; Misbach et al. 2014; Lavoue et al. 2014; Brenten et al. 2016).

The Greyhound is a commonly mentioned breed in which deviations of some parameters from the reference ranges have been identified. Changes in red blood cell parameters, including higher erythrocyte counts, haematocrit values, and haemoglobin concentrations, along with lower leukocyte and platelet counts have been documented in Greyhounds (Steiss et al. 2000; Campora et al. 2011). Greyhounds also exhibit higher plasma creatinine concentrations than other breeds, presumably due to their larger muscle mass (Freeman et al. 2003). With the observed variations in Greyhounds, an examination was conducted of other breeds belonging to the sighthound group as well. Greyhounds were found to be most similar to Whippets, while significant differences were identified between the other sighthounds. Thus, the reference ranges established for Greyhounds do not apply to other sighthounds. Ideally, each breed within this group should have its reference ranges for haematological and biochemical parameters (Uhrikova et al. 2013). It was subsequently demonstrated that the discrepancies were not only related to haematological and biochemical variables but also to those measured in urine. The findings revealed that Greyhounds exhibit a heightened urinary creatinine concentration compared to non-sighthound breeds, which could also affect the urinary protein-to-creatinine ratio (UPC) (Liffman et al. 2020).

The objective of this study was to assess the concentration of protein and creatinine, as well as their ratio, in the urine of sighthound breeds (FCI group 10) and to contrast these findings with the values of non-sighthound breeds.

## MATERIAL AND METHODS

### Animals and urine samples

Sighthound owners were contacted directly, through kennel clubs or social networks, requesting a urine sample. Samples from dogs of other breeds were primarily provided by students and staff of the University of Veterinary Sciences Brno (Czech Republic). Some were obtained during routine preventive health checks in private practice. Only samples from clinically healthy dogs were included in the study. Good health was determined based on a completed medical history form, the absence of clinical signs of disease, and exclusion from medication except for regular vaccination and deworming.

Immediately following collection, urine specific gravity was measured using a hand-held refractometer (RUR2-ATC; Bellingham + Stanley, Tunbridge Wells, UK) and a diagnostic dipstick (HeptaPHAN; Erba Lachema, Brno, Czech Republic) was used to evaluate the chemical properties of the sample (pH, protein, glucose, ketone bodies, bilirubin and blood/haemoglobin). Samples exhibiting a positive reaction for blood/haemoglobin were excluded from subsequent analysis. A total of 309 canine urine samples were obtained. Following diagnostic dipstick testing, 28 samples were discarded due to the presence of blood/haemoglobin (23) or a small sample volume (5).

A complete analysis was thus performed on the remaining 281 samples. A total of 191 samples from 11 different sighthound breeds were included in the study [Whippet (*n* = 50), Saluki (*n* = 31), Spanish Greyhound (*n* = 27), Borzoi (*n* = 15), Greyhound (*n* = 21), Italian Greyhound (*n* = 18), Scottish Deerhound (*n =* 8), Afghan Hound (*n* = 7), Irish Wolfhound (*n* = 6) Azawakh (*n* = 3), Sloughi (*n* = 3), Magyar Agár (*n* = 1)]. The control group comprised 90 urine samples from dogs of various non-sighthound breeds. The characteristics of both groups are presented in Table 1.

**Table 1:** Characteristics of sighthound and non-sighthound groups

		Sighthound (*n =* 191)	Non-sighthounds (*n* = 90)
Females total		87	48
	intact	58	28
	spayed	29	20
Males total		104	42
	intact	78	37
	castrated	26	5
Mean age (years)		3.7	4.4
Range		6 weeks – 14 years	10 weeks – 12 years

**Table 2:** The list of sighthound and non-sighthound breeds with body weight up to 15 kg

Sighthound breeds		Non-sighthound breeds
Whippet (50×) Italian Greyhound (18×)		Miniature Schnauzer (3×)
		Crossbreed, French Bulldog, Sheltie (2×)
		Border Terrier, Chihuahua, Coton de Tulear, Dachshund, English Miniature Bull Terrier,
		German Spitz, Jack Russel Terrier, Maltese Dog, Miniature Pincher, Parson Russel Terrier, Pug (1×)
Total	68	20

### Laboratory analysis

Urine protein concentration was determined by spectrophotometry, employing the benzethonium chloride method. Urine creatinine concentration was determined using the Jaffe method. Both analyses were conducted on an automated biochemical analyser Konelab 20i (Thermo Fisher Scientific, Waltham, MA, USA) with commercial kits [Abott for urine protein (Urine/CSF Protein; Abbott GmBH, Wiesbaden, Germany), Biovendor for creatinine (Creatinine; Biovendor, Brno, Czech Republic)]. For the evaluation of urine creatinine concentration, the samples were diluted (50 μl of urine sample + 2 450 μl of ultrapure water). The UPC was then calculated according to the following formula: UPC = [urinary protein concentration (mg/l)/10] / [urinary creatinine concentration (mmol/l) × 11.3].

### Statistical analysis

Statistical analysis was performed using the Unistat for Excel v6.5 software (London, UK). The threshold for statistical significance was set at *P* < 0.05 for all statistical analyses. The assumptions of normality and homogeneity of variance were analysed using the Shapiro-Wilk and Levene tests, respectively. As the assumptions of normality were not satisfied, the Kruskal-Wallis ANOVA, followed by Dunn’s post-hoc test, was performed. The protein and creatinine concentrations in urine and UPC were compared between the entire groups of sighthounds and non-sighthounds. Subgroups within the sighthound group were subsequently created based on breed; only subgroups with more than ten individuals were included in further statistical comparisons. The control group was then divided into two parts. One subgroup consisted of breeds and crossbreeds with body weights up to and including 15 kg (*n* = 20) and was compared with Whippets and Italian Greyhounds. The other subgroup consisted of individuals with body weights greater than 15 kg (*n* = 70) and was compared with Saluki, Greyhounds, Spanish Greyhounds, and Borzois (Tables 2, 3).

**Table 3:** List of sighthound and non-sighthound breeds with body weight over 15 kg

Sighthound breeds		Non-sighthound breeds
Saluki (31×) Spanish Greyhound (27×) Greyhound (21×) Borzoi (15×)		Crossbreed (13×)
		Border Collie (6×)
		German Shepherd (5×)
		Czechoslovakian Wolfdog (4×)
		Airedale Terrier, Belgian Shepherd, Collie, Golden Retriever (3×)
		Australian Shepherd, English Cocker Spaniel, Labrador Retriever, Small Münsterlander (2×)
		Alaskan Malamute, English Springer Spaniel, Australian Cattle Dog, Bearded Collie, Bernese Mountain Dog, Brittany, Dalmatian, Dobermann Pinscher, Entlebucher Mountain Dog, Eurohound, Flat Coated Retriever, Siberian Husky, Chesapeak Bay Retriever, Bohemian Shepherd, German Shorthaired Pointer, American Pit Bull Terrier, Portuguese Water Dog, Rhodesian Ridgeback, Rottweiler, Samoyed, Greater Swiss Mountain Dog, Weimaraner (1×)
Total	94	70

## RESULTS

When comparing the two groups of dogs (sighthounds and non-sighthounds), no difference was found in urine specific gravity (mean ± standard deviation: 1.042 ± 0.014 and 1.042 ± 0.013, respectively; *P* = 0.754). The sighthounds exhibited a significantly lower urinary protein concentration (mean ± standard deviation: 248.8 ± 275.3 mg/l and 299.8 ± 321.1 mg/l, respectively; *P* = 0.045) and a highly significantly higher urinary creatinine concentration (mean ± standard deviation: 23.0 ± 11.5 mmol/l and 17.5 ± 8.5 mmol/l, respectively; *P* = 0.000) compared to the entire control group. Upon subsequent calculation of the UPC, it was observed that sighthounds demonstrated significantly lower values than non-sighthounds (mean ± standard deviation: 0.13 ± 0.25 and 0.18 ± 0.22, respectively; *P* = 0.000).

**Figure 1:**
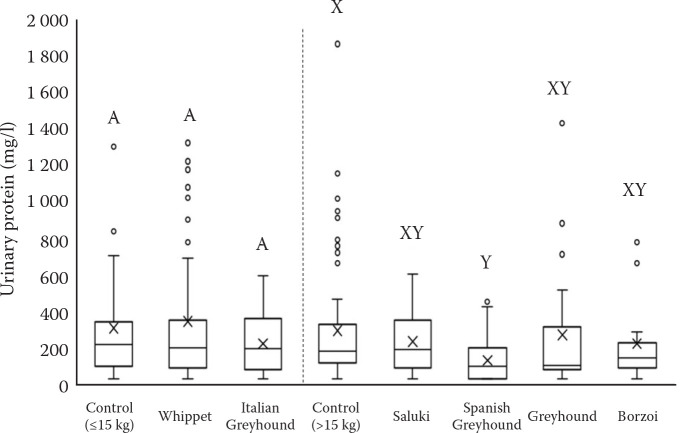
Results of urinary protein concentration in individual sighthound breeds categorised by weight and corresponding control groups Within each boxplot, the horizontal lines denote the median values; the cross indicates mean value, and the boxes extend from the 25^th^ to the 75^th^ percentile of each group’s distribution of value. The lower and upper whiskers indicate the smallest value within 1.5 times the interquartile range below the 25^th^ percentile and the largest value within 1.5 times the interquartile range above the 75^th^ percentile, respectively. Dots located outside of the minimum or maximum whisker represent outliers. Significant differences (*P* < 0.05) among groups are indicated by different alphabetical superscripts. Separate comparisons were performed for small and large breeds, including controls (A, B for dogs with body weight below 15 kg; X, Y for dogs with body weight above 15 kg)

**Figure 2:**
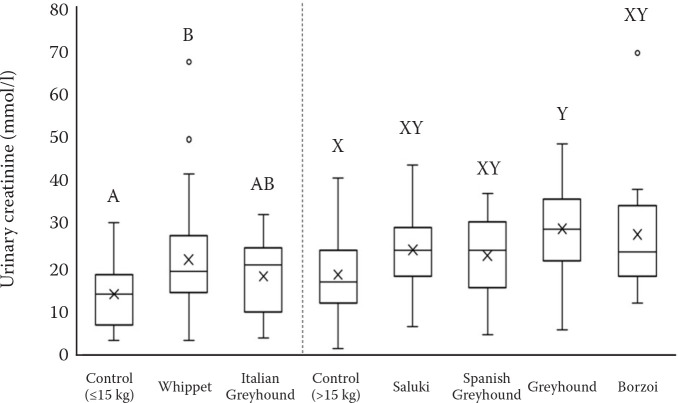
Results of the urinary creatinine concentration in individual sighthound breeds categorised by weight and corresponding control groups Within each boxplot, the horizontal lines denote median values; the cross indicates the mean value, and the boxes extend from the 25^th^ to the 75^th^ percentile of each group’s distribution of value. The lower and upper whiskers indicate the smallest value within 1.5 times the interquartile range below the 25^th^ percentile and the largest value within 1.5 times the interquartile range above the 75^th^ percentile, respectively. Dots located outside of the minimum or maximum whisker represent outliers. Significant differences (*P* < 0.05) among groups are indicated by different alphabetical superscripts. Separate comparisons were performed for small and large breeds, including controls (A, B for dogs with body weight below 15 kg; X, Y for dogs with body weight above 15 kg)

**Figure 3:**
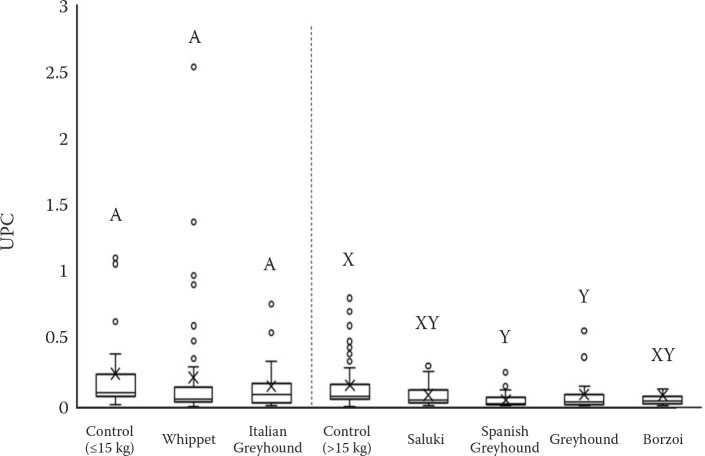
Results of the urinary protein to creatinine ratio (UPC) in individual sighthound breeds categorised by weight and corresponding control groups Within each boxplot, the horizontal lines denote median values; the cross indicates the mean value, and the boxes extend from the 25^th^ to the 75^th^ percentile of each group’s distribution of value. The lower and upper whiskers indicate the smallest value within 1.5 times the interquartile range below the 25^th^ percentile and the largest value within 1.5 times the interquartile range above the 75^th^ percentile, respectively. Dots located outside of the minimum or maximum whisker represent outliers. Significant differences (*P* < 0.05) among groups are indicated by different alphabetical superscripts. Separate comparisons were performed for small and large breeds, including controls (A, B for dogs with body weight below 15 kg; X, Y for dogs with body weight above 15 kg)

A comparison of individual sighthound breeds with their corresponding control groups revealed lower protein concentrations in Spanish Greyhounds (*P* < 0.05), higher urinary creatinine concentrations in Whippets (*P* < 0.05) and higher creatinine concentrations in Greyhounds (*P* < 0.01) (Figures 1, 2). Upon assessment of UPC, lower values were identified in Greyhounds and Spanish Greyhounds compared to the control group (Figure 3).

## DISCUSSION

The urine protein-to-creatinine ratio is the accepted gold standard for quantifying proteinuria in veterinary medicine. The measured urinary protein concentration is related to the observed urinary creatinine concentration, the production of which should be at a constant rate in the body. Filtration by the glomeruli is not limited, and resorption or secretion by the tubules is negligible. Consequently, the creatinine concentration in urine depends primarily on the volume of urine produced (Roura et al. 2017). Creatinine is a small molecule formed as a product of muscle metabolism. Its serum concentration is mainly influenced by the amount of muscle mass and exhibits a positive correlation with body weight (Craig et al. 2006). Therefore, it is reasonable to posit that body weight influences also urinary creatinine concentration. For this reason, the control group was divided into two parts. The first subgroup consisted of individuals with a body weight up to and including 15 kg. These individuals were compared with small sighthounds (Whippets and Italian Greyhounds). The second subgroup consisted of representatives of breeds with a body weight greater than 15 kg and was used in comparison with larger sighthounds (Saluki, Greyhound, Spanish Greyhound and Borzoi breeds).

The impact of urinary blood contamination has been assessed in several previous studies, with findings reporting ambiguous results that may have potentially been influenced by differences in methodology. Bagley et al. (1991) reported that urinary blood contamination and experimentally induced cystitis were associated with an increased UPC. However, a limitation of the cited study is that the urinary protein concentration was detected by a relatively insensitive method (protein precipitation with trichloroacetic acid) and a value of 2.0 was set as the upper limit of the reference range for UPC. According to Vaden et al. (2004), microscopic haematuria does not increase urinary albumin concentration, and macroscopic haematuria does not increase UPC. However, a recent study has demonstrated that the UPC value of urine samples devoid of blood contamination is significantly lower than that of samples with blood contamination, irrespective of the colour of the sample. This finding may potentially lead to confusion regarding the classification according to the guidelines of the International Renal Interest Society (Vientos-Plotts et al. 2018). For this reason, the decision was made not to include samples with blood detected using diagnostic dipsticks in the present study.

The results of this study indicate that variations in urinary creatinine or protein concentrations lead to different UPC values in sighthound breeds compared to other breeds. To date, the only breed that has been subject to investigation in this area is the Greyhound. Liffman et al. (2020) observed higher urinary creatinine concentrations in Greyhounds, however, the mean UPC value did not differ from that of dogs of other breeds of similar age, sex, and weight. The results for the Greyhound group are consistent with these findings. The reference interval in the cited study was set at 0.037–0.43 and 0.036–0.23 when outliers were excluded. Although these values are lower than those reported for the general dog population, the authors concluded that this is not a clinically significant difference.

The elevated urinary creatinine concentration observed in sighthounds is probably most likely attributable to a corresponding increase in plasma creatinine concentration. Other factors were also considered, namely, the active tubular creatinine secretion and a higher glomerular filtration rate. Previous studies have demonstrated that tubular creatinine secretion is of negligible importance in dogs (KuKanich et al. 2007). The glomerular filtration rate in greyhounds has been investigated in several studies. However, given the inconsistency of their findings, further research is required (Hill et al. 2007; Mesa-Sanchez et al. 2012). Nevertheless, it is notable that other breeds of sighthounds also exhibit higher plasma creatinine concentrations. Whippets display haematological and biochemical parameters similar to those observed in Greyhounds (Uhrikova et al. 2013). Therefore, it was anticipated that a statistically significant elevation in urinary creatinine concentration would be observed in Whippets, as was corroborated by the findings of our study. The issue pertains to the lower urinary protein concentration in Spanish Greyhounds. This subgroup numbered 27 dogs, 9 of which (33%) showed urinary protein concentrations below the detection limit. This was the youngest subgroup of all sighthounds. To gain a more accurate assessment, it would be beneficial to test a larger group of dogs of varying ages and possibly employ a more sensitive method.

Despite the discovery of statistically significant discrepancies in UPC values between sighthounds and non-sighthounds, in addition to lower UPC values observed specifically in Greyhounds and Spanish Greyhounds, these variations are probably not clinically important. Thus, breed affiliation is unlikely to influence the diagnosis of proteinuria by UPC in sighthounds.
